# Confidentiality and treatment decisions of minor clients: a health professional’s dilemma & policy makers challenge

**DOI:** 10.1186/2193-1801-3-320

**Published:** 2014-06-26

**Authors:** Margot Karen Jackson, Katharina Kovacs Burns, Magdalena S Richter

**Affiliations:** Faculty of Health and Community Studies, MacEwan University, 10700-104 Avenue, Edmonton, AB T5J 4S2 Canada; Faculty of Nursing, University of Alberta, 1405-87 Avenue, Edmonton, AB T6G 1C9 Canada; Interdisciplinary Health Research Institute, Health Sciences Council, University of Alberta, 11405-87 Avenue, Edmonton, AB T6G 1C9 Canada

## Abstract

Issues relating to confidentiality and consent for physical and mental health treatment with minor clients can pose challenges health care providers. Decisions need to be made regarding these issues despite the absence of clear, direct, or comprehensive policies and legislation. In order to fully understand the scope of this topic, a systemic review of several pieces of legislation and guidelines related to this topic are examined. These include the: *Canadian Human Rights Act*, *Children’s Rights: International and National Laws and Practices, Health Information Act, Gillick Competence and Medical Emancipation, Freedom of Information and Protection of Privacy Act, Child, Youth and Family Enhancement Act, Common Law Mature Minor Doctrine, and Alberta Health Services Consent to Treatment/Practice(s) Minor/Mature Minor*. In order to assist health professionals with decisions regarding confidentiality and treatment with minor clients a case study and guide for decision-making is also presented.

## Introduction

Working with minor clients can pose many challenges for health care providers. This is particularly true in relation to issues of confidentiality and consent for treatment for physical and mental health concerns. This population is “…especially difficult since they straddle the conceptual and definitional divide between childhood and adulthood” (Ferguson, 2004, p.3). Many youth may have the maturity to decide the course of their health related decisions however their right to confidentiality rests in the hands of those who are providing their care. This holds true as current legislation merely provides guidelines for health care professionals to determine if the minor is capable of making informed choices and what issues are to remain confidential. The issue of confidentiality in minors leads into other areas of interest as well, including whether or not a child or adolescent can refuse or consent to their own treatment without the consent of their parent or guardian. In conducting a literature review on this subject area, it becomes apparent there exist two separate but related issues. The first being that of confidentiality of a minor client’s health information, and the second being a minor client’s ability to consent to medical treatment.

Several circumstances exist in which this may pose a problem for health professionals, such as a minor requesting an abortion or refusing to receive blood products due to religious beliefs. Making a decision in these situations is not simply deciding whether to provide treatment or not. There are a number of considerations to take into account and health professionals must unfortunately weigh them in the absence of clear, direct policies and legislation.

In order to fully understand the scope of this topic, several pieces of legislation and guidelines must be examined. These include the: Canadian Human Rights Act, Children’s Rights: *International and National Laws and Practices, Health Information Act, Gillick Competence and Medical Emancipation, Freedom of Information and Protection of Privacy Act, Child, Youth and Family Enhancement Act* and the *Common Law Mature Minor Doctrine*. This paper will conclude with a case study of a minor client that includes: a discussion of the concerns and challenges of those involved in attending to the care needs of the client, the processes and policies or guides used to help inform the decisions made for care and treatment, and some recommendations for health professionals and health policy decision makers.

### Essential context: understanding the terminology

Prior to reviewing current legislation and guidelines and discussing the case study, there are several terms needing to be defined for further understanding of this subject area. First, the term “youth” refers to those individuals who are between 14–18 years of age, which is in accordance with Alberta Health Services ([n.d].). Second, “confidentiality” is understood to be any information that is shared in confidence within the health care setting or within the health care relationship between patient and caregiver (Tan et al. 2007). Confidentiality is a key element in providing health care to individuals. Patients’ understanding that their personal health information will be kept in confidence, and used only to benefit their care and treatment, is fundamental to health care in Canada. Furthermore, confidentiality is the basis to establishing a therapeutic relationship between health care provider and client, which can lead to more successful treatment and outcomes (Tan et al., 2007). The terms of confidentiality become more complex when addressing the health concerns of children and youth, and bring forward the need for assessment of *capacity. Capacity* of understanding is defined legally as the ability to make treatment decisions. This term is similar to that of *competence* which is used more frequently in clinical settings to determine if an individual can make decisions for themselves as in the case of mental illness (Tan et al. 2007). Finally, the legal term of “informed consent” is described as:

“…the informed agreement of a Patient or Alternative Decision-Maker (if applicable) prior to the Patient undergoing a Treatment/Procedure(s) after being provided with the relevant information about the Treatment/Procedure(s), its risks and alternatives and the consequences of refusal” (Alberta Health Services, 2010, p.9).

## Methodology

Information for this paper was gathered from searches citing the keywords “confidentiality, health information, policy, youth, children, mature minor” in the CINAHL and MEDLINE databases, the Canlii and Quicklaw websites and Google Scholar. As well as, applicable legislation was gathered and obtained from the Alberta Government Queens Printer and through personal communication with Alberta Health and Wellness Information Policy and Compliance Unit. A summary of each document has been completed to provide some context for what information and legislation is available to support or challenge the decisions which health professionals must make regarding the confidentiality of information and treatment of minor clients in Alberta, and to some extent, in other parts of Canada. Canadian guidelines are often used as there are no provincial specific guidelines for many areas related to the care and treatment of minor clients.

### Legislation and related documents

#### ***Code or ethics for registered nurses (Codes and Declarations***^1998^***)***

In the Code of Ethics for Registered Nurses , confidentiality is addressed by stating that “Nurses safeguard the trust of clients that information learned in the context of the professional relationship is shared outside the health care team only with the client’s permission or as legally required” (p. 70). The nurse is required to keep clients’ information confidential and only disclose if there is potential for serious harm to the client or legal grounds for doing so. Unfortunately, there are no specific guidelines for information relating specifically to children and youth in this document. It can therefore be assumed that these rights of confidentiality apply to persons of all ages. However, additional legislation and policies must be addressed for a nurse to make an informed decision.

### Consent: a guide for Canadian physicians (College of Physicians and Surgeons of Ontario, 2002)

The Canadian Medical Protective Association has created guidelines for physicians to follow in regards to confidentiality and consent for treatment. Within these guidelines, the age of consent is discussed as a minor who may have the capacity necessary to make treatment decisions. The College of Physician and Surgeons of Ontario (2002) state that “The determinant of capacity in a minor has become the extent to which the young person’s physical, mental, and emotional development will allow for full appreciation of the nature and consequences of the proposed treatment, including the refusal of such treatments” (p.5). In terms of chronological age, the Courts in Alberta have not established a set age for maturity; however “the threshold for recognition of maturity by the Courts is at least sixteen years and none have recognized individuals younger than fourteen years” (College of Physicians and Surgeons of Alberta, 2006, p. 1). It is also important to consider that the Alberta Ministry of *Children’s Services* recognizes the age of twelve as being old enough to discuss and seek a child’s opinion on treatment decisions. Lastly, if a patient is deemed a mature minor, the physician has a duty to keep the personal health information of that patient confidential in most circumstances as stated in section 104 of the *Health Information Act.* (College of Physicians and Surgeons of Alberta, 2006).

### Canadian human rights act (Minister of Justice, n.d.)

The essence of the Canadian Human Rights Act (Minister of Justice, n.d.) is to ensure all individuals have equal and fair opportunities to establish their lives without being prevented in doing so by discriminatory practices (Canadian Human Rights Act, Sec.1). The Canadian Human Rights Act (Sec.3) continues to describe the prohibited grounds of discrimination as race, ethnic origin, religion, age, sex, family status, sexual orientation, colour, marital status, or disability. For the purposes of our discussion it is important to note that age is included as one of the prohibited grounds for discrimination. As a result, confidentiality of minors cannot be simply dismissed due to age alone. The health care practitioner must recognize that children have basic rights and should consider maintaining confidentiality under specific circumstances.

### Children’s rights: international and national laws and practices (Law Library of Congress, 2007)

In 1989 the United Nation Convention on the Rights of the Child (UNCRC) was developed as part of the celebration of the United Nations Year of the Child (Charles-Edwards, and Brotchie, 2005). The UNCRC is child centered and has introduced new thinking in relation to the concept of family. According to the UN convention “families and family life are important …because children need families rather than because adults have the right to have a family” (p.41). Charles-Edwards and Brotchie (2005) suggest that this idea differs from the notion that the family has a right to privacy from the state and places greater worth on the child and their needs as was previously seen in the *European Convention on Human Rights and Fundamental Freedoms* (ECHR). The UNCRC differs from the ECHR in that the child is seen as an individual not purely as a part of a family whose decisions are dictated by the parents. These ideas relate to concerns identified by UK Law and the European Court of Human Rights which felt there was concern about how decisions would be made for children (Charles-Edwards & Brotchie, 2005).

In Canada, only those who have reached the age of majority can be free of parental control, exercise full civil rights, and have the capacity to enter into legal contracts (Law Library of Congress, 2007). Despite this law, youth often engage in health-related treatment decisions without parental consent and do have the capacity to understand their decision. Tan, et al. (2007) state that “Research has shown that children acquire the capacity to make treatment decisions for appropriately-simplified treatment information by the age of approximately nine years, and for adult-level treatment by the age of 14 years” (p.196). *Children’s Rights in Canada* (Law Library of Congress, 2007) do not discuss the topic of health care decisions and confidentiality however, they do state that a juvenile may be sentenced to an adult sentence in court if “a judge finds that a youth sentence would not be sufficient” (p.59). Why is it that a youth can be seen as responsible in one area but not in another? Can a youth not make a decision about their health and confidential matters if they can be tried as an adult in the judicial system? It is always important to consider every aspect related to a child and their family when making decisions related to confidentiality. A fine line exists here, as subjectivity plays a role in a health provider’s interpretation of a given situation.

### Freedom of information and protection of privacy act (Government of Alberta, 2000)

The basic objectives of the Freedom of Information and Protection of Privacy Act (FOIP) are to “ensure that public bodies are open and accountable to the public by providing a right of access to records” and “to protect the privacy of individuals by controlling the manner in which public bodies collect, use and disclose personal information” (Government of Alberta, 2000, p.1). As a general rule the FOIP Act does not apply to health information (FOIP, sec. 4.1.U). There may however be some circumstance where health information and personal information may be entwined, in which case both must be kept in confidence. The health related information would be subject to regulation under the *Health Information Act* and the personal information would be subject to privacy under FOIP. As a health professional, information shared from a patient would fall under the jurisdiction of the Health Information Act as the context of the communication is related to health concerns.

### Health information act (Government of Alberta, [n.d].)

In Alberta, the Health Information Act (HIA) establishes rules for collection, use, disclosure and protection of health information primarily in the publically funded system. The HIA requires health providers to collect, use and disclose the least amount of information they need to provide care and treatment. Furthermore, the HIA takes steps to ensure confidentiality of health information for all individuals. Section 60(1)(a) of the HIA state that “A custodian must take reasonable steps in accordance with the regulations to maintain administrative, technical and physical safeguards that will protect the confidentiality of health information that is in its custody or under its control and the privacy of the individuals who are the subjects of that information”. There is no mention in this section regarding confidentiality for those under the age of majority who wish to keep their health information from being shared with a parent or guardian. There are however two sections of the HIA that pertain to children and youth, one directly and the other indirectly. First, Section 35 (subsections c, m, n,) of the HIA discusses the disclosure of diagnostic, treatment, and care information. Under this section it is stated that:

A custodian may disclose individually identifying diagnostic, treatment and care information without the consent of the individual who is the subject of the information:

(c) to family members of the individual or to another person with whom the individual is believed to have a close personal relationship, if the information is given in general terms and concerns the presence, location, condition, diagnosis, progress and prognosis of the individual of the day on which the information is disclosed and the disclosure is not contrary to the express request of the individual.
(m) to any person if the custodian believes, on reasonable grounds, that the disclosure will avert or minimize an imminent danger to the health or safety of any person.
(n) if that individual lacks the mental capacity to provide consent and, in the opinion of the custodian, disclosure is in the best interests of the individual.

In accordance with this direction, a child or youth who has not formally asked a health care professional to keep their information in confidence, may have their treatment or care disclosed to their parent or guardian. However, if the child youth does ask for an express request of confidentiality, the health care provider must consider this request. Furthermore, the health care provider can disclose information if he/she feels that the young person is at risk or cannot make an appropriate decision for his/her treatment and care. This leads to the second piece of the HIA that directly applies to those under age 18 years. Section 104(1) b states: “Any right of power conferred on an individual by this Act may be exercised if the individual is under 18 years of age and understands the right of power and the consequences of exercising the right of power, by the individual” (Government of Alberta, [n.d]., p.59). Consequently, if a nurse or health care provider feels that the child or youth understands the nature of their decision and is capable of making personal decisions then they can proceed with treatment or care. The HIA does not explicitly provide any specific ages of a child or youth for the health care provider to use a guide for decision-making. It would appear that each case should be examined on an individual basis and decisions surrounding that case be considered carefully and professionally by those involved. Charles-Edwards and Brotchie (2005) outline three situations in which confidentiality of minors can be breached:

1. When the child him/herself gives you permission to tell someone else. This allows the child to keep some control over his/her private information
2. Where it is required by law, such as in the case of a notifiable disease
3. Where it is your professional judgment that is in the public’s or the child’s best interests. This is the most relevant situation and is of course when most difficulties tend to arise (p.40).

In Canada, the HIA does not outline specific circumstances or a chronological age to where a young person can retain their confidentiality of health related information. This decision is placed on the health care provider based on the specific circumstances and details surrounding their patient.

### Gillick competence and medical emancipation

In Britain, sexual health matters are routinely kept confidential and have led to legislation surrounding the rights of young people under 16 years receiving confidential medical, sexual and contraceptive advice (Jenkins, 2004). Youth ages 16–17 years in the U.K. already have rights equal to adults concerning medical treatment and confidentiality (Jenkins, 2004). A central case in U.K. law related to the rights of children less than 16 years of age is Gillick v. West Norfolk, 1985 (Griffith, 2013). In this case, Victoria Gillick requested that her local health authority not provide any services to her daughters, who were under the age of 16 years, without her consent. It was determined in the House of Lords that the health authority “could give medical advice to young people under the age of 16 under certain conditions” (Jenkins, 2004, p.2). Furthermore, the child or youth’s right to confidentiality is dependent upon their maturity and level of understanding not simply a chronological age. This court decision has led to the term *Gillick competent* (Palmer & Gillespie, 2014) when referring to a minor client’s capacity to make personal health care decisions independently. In order to ascertain if a minor is Gillick competent, they are assessed for both maturity and intelligence. This would include gaining an understanding of the minor’s ability to manage family and peer pressure, fear, and information, as well as their ability to weigh the benefits and risks of medical treatment (Griffith, 2013). It may be presumed that if a minor has been deemed *Gillick Competent*, their health related information is to be kept in confidence from their parent or guardian. The case of Gillick v. West Norfolk has been upheld by the Supreme Court of Canada as seen in the court case A.C. *v*. Manitoba, 2009.

In the United States, some states do not require a parent or guardian to be notified if their child presents with certain medical conditions. These conditions are considered to be medically emancipated and state laws do not require consent of a parent or guardian (Anderson et al. 2006). Conditions that are considered medically emancipated in some states include: contraception, pregnancy and prenatal care, sexually transmitted infections, human immunodeficiency virus, substance abuse, mental health disorders, treatment after sexual assault and pelvic examination (Anderson et al., 2006, p. 56).

### Child, youth and family enhancement act (Province of Alberta, 2014.)

The Child, Youth and Family Enhancement Act states that “a child is in need of intervention if there are reasonable and probable grounds to believe that the survival, security, or development of the child is endangered” (Sec.2, p. 7). There are several reasons outlined in Section 2 of the Act that would warrant intervention including: abandonment, neglect, physical or sexual abuse, refusal to provide the child with essential medical services, unwillingness to protect the child from emotional injury, or subjecting a child to cruel and unusual treatment or punishment. There is an obligation for all individuals in the community to report concerns of child maltreatment and/or related issues to children’s services. As declared in the Act under Section 4(1) “Any person who has reasonable grounds to believe that a child is in need of intervention shall forthwith report the matter to a director”. Additionally, the Human Resources and Skills Development Canada document entitled *Child Welfare in Canada 2000* (Government of Canada, 2000) states that “ The duty to report applies to all persons, even those who are obliged by professional standards or statutes to keep information in confidence” (p.6). This piece of legislation is a foundational guideline for health care professionals when working with children and youth. Any decisions regarding confidentiality and privacy of a client must first be examined in terms of potential harm or risk to the young person involved. Only after these issues have been ruled out, can the health care provider continue to debate the risks and benefits of disclosing confidential health information to a parent or guardian.

### Common law mature minor doctrine (Ferguson, 2004)

In Canada, the Common Law Mature Minor Doctrine addresses the ability of a minor to consent to medical treatment. This doctrine examines the capacity of the minor in regards to decision-making and their “cognitive” capacity in regards to their understanding and appreciation of the proposed medical treatment (Ferguson, 2004). The decision rests upon the health care provider to determine if a youth is a mature minor based upon a thorough assessment of the patient who is under the age of majority and follow their own discretion. Unfortunately, the doctrine does not speak to issues of confidentiality for minors directly. In Alberta, the Child, Youth and Family Enhancement Act has supplanted the mature minor doctrine yet many other provinces in Canada still adhere to its guidelines (Ferguson 2004).

### Alberta health services “consent to treatment/procedures for minor/mature minor” (Alberta Health Services, 2010)

Although there is little guidance in current legislation regarding confidentiality of a minor’s health information, some does exist to direct health care professionals in deciding if a youth is a mature minor and can consent or decline health treatment. Alberta Health Services (AHS) does have a document available titled *Consent to treatment/procedure(s) minor/mature minors* which discusses the consent process for mature minor deemed to have capacity to make treatment decisions. Under section 1.1 of this document, a minor’s capacity is determined by a responsible health care practitioner. The section reads, “A *Patient under the age of eighteen (18) years is presumed to be a Minor Patient without Capacity, unless assessed and determined to be a Mature Minor:*

- *Health Practitioners shall conduct the assessment for a Mature Minor by asking questions in order to determine whether the Minor Patient has the intelligence and maturity to provide consent for a Treatment/Procedure(s) without the input of their Legal Representative*” (Alberta Health Services, 2010, p.2). AHS also encourages the health care professional to document their assessment finding to support their decision as many of the criteria are subjective in nature. According to AHS the definition for a mature minor is “a person aged less than eighteen (18) years, who has been assessed and determined as having the intelligence and maturity to appreciate the nature, risks, benefits, consequences, and alternatives of the proposed Treatment/Procedure(s), including the ethical, emotional and physical aspects” (Alberta Health Services, 2010). Although this AHS document provides this definition and guidelines for practitioners in regards to treatment matters, it does not address *how* to assess a minor for capacity or what criteria constitute being deemed a mature minor. Furthermore, the issue of confidentiality for health information provided by youth is not addressed in this document. As stated in the College of Alberta Psychologist Consent for Minors document (2009) “It is commonly assumed that the mature minor doctrine includes confidentiality owed to a mature minor which therefore, would deny a guardian access to the mature minor’s personal health information. However, the Courts have never declared this to be part of the mature minor doctrine” (p.9).

### Case study

Carla is a 15 year old girl who presents with a treatable sexually transmitted infection for which she is to receive antibiotics. Carla is very worried about her boyfriend and his reaction to this news and how it will affect their relationship. She currently lives with her Mom and younger brother as her parents divorced 2 years ago. Carla and her Mom have been arguing lately as she has broken curfew several times while she was with her boyfriend. She does not wish her Mom to be told of her infection or sexual activity. Carla attends school regularly where she maintains a B average. She plays soccer with her community league team and has many friends at school. A few months ago, Carla was very “stressed out” and cut a few small marks on her upper legs to relieve some of the tension. The marks were superficial and did not require any medical attention. Carla states that she has only self-injured this one time and has no suicide ideation or plan. She is adamant that her mother not be told of her situation^a^.

### Concerns and recommendations

In Alberta, there are many issues or concerns for health professionals who care and treat minor clients such as Carla in this case study. No single or designated policies or legislation exist in this area of confidentiality and treatment decisions of minor clients, specifically to direct or protect health care professionals in their decisions about confidentiality of children, and particularly youth. There are pieces of legislation such as the *Health Information Act* and *Child, Youth and Family Enhancement Act* that outline certain protocols, yet in practice, situations are often subjective and complicated. The consequences of not having policies and procedures to follow directly could be dire as decisions can still have additional consequences for either the client or the health professionals or both, and certainly for parents/families if they are either excluded or included in the decisions and information about the client. As one example, a youth, deemed to have the maturity capacity by a health care provider, decides to have an abortion but she may not be able to mentally cope with the follow up of this decision. This could result in future mental health concerns such as depression and suicidal ideation. As another example, an adolescent female is treated for a sexually transmitted infection and her parents are informed. As a result, she is beaten by her father and kicked out of her home. A case in the Canadian media is that of a 14 year old girl of the Jehovah’s Witness faith receiving blood products against her wishes. The youth may have had the capacity to make this decision but the courts decided that under the *Child, Youth and Family Enhancement Act* it was in her best interests to receive the treatment (Supreme Court of Canada 2009). In this case, the severity of the medical treatment came into play, as this was a proposed life or death situation.

In addition, there is little guidance in current legislation to protect the health care provider from litigation from parents or guardians, as their decision to keep information confidential is often based on subjective assessment information. It rests upon the health care professional to prove that he/she thoroughly assessed the minor for capacity of understanding and provided adequate and sufficient information to warrant an informed consent if treatment is requested.

Another issue is that minor patients should have confidence that they can freely share personal health information with health care professionals who are treating or caring for them, without fear of disclosure. Many youth would not access certain health services (i.e. birth control clinic, sexually transmitted disease clinic) if they knew that all of their information would be shared with their parents. The result could be detrimental to the health and well-being of the youth and lead to further, more serious problems, in the future.

A main concern is the difficult challenge which health care providers have in deciding whether or not a youth has the capacity to understand their health care issues and what information should be kept in confidence. As health care professionals want to provide the best care possible for their clients, a guide is essential to assist them in their difficult decision making situations. As a result of this enquiry into the existing legislation and related documents and finding the lack of sufficient guides for health professionals, a guide for assisting them in reaching this decision has been created. This guide is adapted from Tan et al. (2007) “Confidentiality Decision-Making Algorithm for Children and Adolescents” and the Government of Alberta (2007) document “Information Sharing for Human Service Providers in the Alberta Public Sector” (Government of Alberta) and provides a series of steps which health professionals may follow in order to make the most appropriate or ‘best’ decision based on the evidence gathered with the minor client as well as the existing policies and options available. The details of this guide is presented as follows:

**Youth Confidentiality Guidelines**

Step 1: Gather all relevant information and assess physical and psychological state. Explain limits of confidentiality
Does the information shared…
  - Require intervention under the Child, Youth and Family Enhancement Act?
  - Identify a communicable disease and require disclosure based on the Mandatory Testing and Disclosure Act?
  - Pose imminent danger or harm to the health or safety of the individual or another person?
  - Have a Court Order applied?
  ***If yes****…*Must report to appropriate authorities
  ***If no****…*Encourage minor to disclose to parent/guardian
    -If the minor agrees… disclose the minimum required.
    If the minor disagrees this is now an express request under the HIA section 35(c)…proceed to *Step 3.*
Step 3: Determine capacity. This includes assessment of the following factors:
  - Current age of the minor
  - Level of intelligence (cognitive abilities)
  - Decision making capability (presence of cognitive delays, mental illness, drug usage)
  - Ability to understand informed consent
  - Nature and/or seriousness of condition or illness
  - Living independently, little or no contact with parents or guardian, or are a parent themselves
  *If deemed a mature minor*all information is kept confidential*
  *If not deemed a mature minor*allow the youth to choose with whom they wish to disclose information and only disclose the minimum required*

### Appling the guide in the discussion of the case study

The proposed guidelines can be applied directly by the health care provider who is caring for and treating a minor client. For the care and treatment of 15 year old Carla, the question of confidentiality becomes complex because of the self-inflicted harm. Is this something that should be kept confidential or are there family and health care professionals in mental health who should be notified about Carla and circumstances as well as her current health condition? How can the Youth Confidentiality Model including the determination of mature minor status be applied in this case?

Step 1 in the model would consider what relevant information could be gathered from Carla regarding her physical and psychological state. Another thing to determine is what relevant information can be used from the various current policies and legislation to guide decisions of health care providers. This is actually included in Step 2 along with other tasks. So, based on findings from step 1 and 2, does Carla need to be reported to the appropriate authorities or specialists? Or, should she be encouraged to disclose to her parents/guardians? In this situation, Carla’s STI is considered a notifiable disease by the Public Health Agency of Canada and is reported without her parent’s knowledge or consent (Alberta Health and Wellness, 2013). As well, Carla’s boyfriend needs to be notified that he has been a contact of an individual with an STI and seek the appropriate treatment however, this can be done without using Carla’s name. The decision was made to encourage Carla to share her situation with her parents but she has refused. This is now an *express request* under the HIA. Continue to Step 3 of the guide.

Applying Step 3 and the six factors to determine Carla’s capacity – her age, level of intelligence, decision making capacity, serious health care related decisions, informed consent, and current living situation. From this Carla could be either deemed a mature minor (all information is kept confidential), or not deemed a mature minor (allow the youth to choose with whom they wish to discuss information and only disclose the minimum required). The decision made in this step is to deem Carla a mature minor and keep all of her information confidential as she is 15 years old, has above average intelligence, maintains a support system, understands informed consent, has an easily treatable infection, understand the treatment modality, and has sought treatment independently. This being said, the health care professional must document their decision to deem Carla a mature minor and follow up with appropriate referrals and treatments.

## Conclusion

Health care professionals have a legal and ethical obligation to keep their patients’ health related information confidential. This obligation is quite clear for adults yet can become convoluted for children and youth, despite the potential that a youth has developed “sufficient intelligence and comprehension to appreciate the nature and consequences of medical treatment” (College of Alberta Psychologists, 2009, p.1). Some legislation does exist in Canada that can serve as a tentative guide for health care workers when faced with dilemmas over confidentiality in young people. Although referral to the *Child, Youth and Family Enhancement Act, Health Information Act, Canadian Human Rights Act* and the *Common Law Mature Minor Doctrine* may help to clarify some ambiguous areas, it would appear that a direct policy on this matter does not yet exist. As a result, children, youth, their families and health care professionals could benefit from the establishment of a policy related to this subject matter. In the absence of policy, a guide for health professionals to use within their practice would prove beneficial. Such a guide has been presented in this paper and applied to one specific case study. The guide warrants further piloting with health professionals and health policy makers who share the concerns about the care and treatment of minor clients. Policy makers have an opportunity to understand the complexity of the challenges that are not only medical decisions but also ethical and legal. Without a policy to more clearly guide health professionals and health administrators in making the ‘right’ informed decision, and to clearly align the minor client in the context of the law and parent/family rights, the decisions made will continue to have questions and consequences for all concerned.

## Endnote

^a^Although this case study is fictitious, it demonstrates the complex issues and situations faced by health care professionals.

## References

[CR1] (2013). Mandatory Testing and Disclosure Act.

[CR2] (2010). Consent to Treatment/Procedure(s) minor/mature minor.

[CR3] (n.d). Consent to Treatment/Procedure(s) minor/mature minor.

[CR4] Anderson SL, Schaehter J, Brosco JP (2006). Adolescent patients and their confidentiality: Staying within legal bounds. Contemporary OB/GYN.

[CR5] Charles-Edwards I, Brotchie J (2005). Privacy: what does it mean for children’s nurses?. Pediatr Nurs.

[CR6] Codes and Declarations (1998). The code of ethics for registered nurses. Nurs Ethics.

[CR7] (2009). Consent of minor patients.

[CR8] (2006). Consent for Minor Patients.

[CR9] (2006). Consent to medical treatment.

[CR10] Ferguson L (2004). The end of an age: Beyond age restrictions for minors’ medical treatment decisions.

[CR11] (2000). Freedom of Information and Protection of Privacy Act. Alberta Queen’s Printer.

[CR12] (2007). Information sharing for human service providers in private sector organizations.

[CR13] (n.d). Health Information Act., pp.

[CR14] (2000). Child Welfare in Canada 2000.

[CR15] Griffith R (2013). Nurses must be more confident in assessing Gillick competence. Br J Nurs.

[CR16] Jenkins P (2004). A parent’s right to know. Counsell Psychother J.

[CR17] (2007). Canada children’s rights: International and national laws and practices.

[CR18] (n.d.). Canadian Human Rights Act.

[CR19] Palmer R, Gillespie G (2014). Consent and capacity in children and young people. Archives of Disease in Childhood-Education and Practice.

[CR20] (2014). Child Youth and Family Enhancement Act.

[CR21] (2009). Decisions-A.C. v. Manitoba (Director Child and Family Services).

[CR22] Tan JOA, Passerini GE, Stewart A (2007). Consent and confidentiality in clinical work with young people. Clin Child Psychol Psychiatr.

